# Proteomic insights into nematode-trapping fungi *Arthrobotrys oligospora* after their response to chitin

**DOI:** 10.2478/jvetres-2025-0005

**Published:** 2025-02-25

**Authors:** Jiahua Zhang, Lixiang Wei, Huimei Zhang, Xixi Ma, Yansen Sun, Ruobing Li, Chengzhi Zhang, Xuepeng Cai, Jun Qiao, Qingling Meng

**Affiliations:** College of Animal Science and Technology, Shihezi University, Shihezi, Xinjiang 832003, China; State Key Laboratory of Veterinary Etiological Biology, Lanzhou Veterinary Research Institute, Chinese Academy of Agricultural Sciences, Lanzhou, Gansu 730046, China

**Keywords:** *Arthrobotrys oligospora*, chitin, chitinase, differentially expressed protein, metabolism

## Abstract

**Introduction:**

Nematode-trapping fungi (NTFs) can produce various chitinases to degrade nematode body wall and eggshell chitin during predation. However, the regulatory mechanisms of their expression of chitinases still remain unclear. The primary objective of this study was to elucidate the differential protein profile of *A. oligospora*, an NTF, in response to chitin.

**Material and Methods:**

Colloidal chitin was added to induce the culture of *A. oligospora*, and the phenotypic differences before and after induction were observed under inverted microscope. The differential proteins before and after mycelium induction were screened by liquid chromatography-tandem mass spectrometry. The differentially expressed chitinase was expressed in *Pichia* yeast, and the recombinant enzyme was incubated with *Caenorhabditis elegans* and its egg suspension to explore its biological activity.

**Results:**

It was found that there was a significant acceleration in the mycelial growth post chitin interaction in *A. oligospora*. A total of 1,124 differentially expressed proteins (DEPs) were identified between the control group (AO-c) and the experimental group (AO-e), with 183 upregulated and 941 downregulated. Gene Ontology analysis revealed that the DEPs acted in various metabolic processes with catalysis and binding functions. Kyoto Encyclopedia of Genes and Genomes analysis associated these proteins primarily with signalling pathways related to glucose metabolism. Three chitinases were significantly modulated among DEPs. Moreover, enzymatic activity assays demonstrated that one of them effectively degraded *C. elegans* and its eggs.

**Conclusion:**

These findings suggest that *A. oligospora* can significantly alter its protein expression profile in response to chitin, thereby facilitating its sugar metabolism and mycelial development. Our study provided new insights into the regulatory mechanisms of nematode predation in *A. oligospora*.

## Introduction

Gastrointestinal nematode disease in livestock, a prevalent parasitic condition in herbivore farming, leads to significant economic losses by causing weight loss, decreased production, diarrhoea and even death in affected animals, thereby impeding the healthy development of the global livestock industry (11, 13, 36). Currently, prevention and control of this disease predominantly rely on chemical anthelmintics (7, 43). However, the prolonged and extensive use of these traditional chemical treatments has led to multiple issues, including nematode resistance, environmental pollution and drug residues in food of animal origin (10, 15). Consequently, there is an urgent need to develop a new type of efficient and environmentally friendly biological control agent to mitigate the shortcomings associated with chemical deworming drugs in managing parasitic nematodes in livestock.

Nematode-trapping fungi (NTFs), as natural adversaries of nematodes, have demonstrated effectiveness in managing gastrointestinal infestations with them in livestock, thereby heightening interest in the biocontrol research of nematode diseases based on NTFs (9, 23, 31, 44). Upon encountering nematodes, NTFs can detect specific signalling molecules and develop specialised structures to ensnare their prey (14, 18, 19, 29). Recent studies show that extracellular protein hydrolases, including the chitinase, serine protease and collagenase produced by predatory fungi, play a crucial role in NTF mechanisms for penetrating, digesting and preying on the nematode cuticle (12, 37, 40). Consequently, these extracellular hydrolases produced by NTFs have been extensively examined as virulence factors during the invasion process.

*Arthrobotrys oligospora*, a representative species of NTF, can produce distinctive predatory fungal rings and adhesive three-dimensional webs, making it a significant microbial resource for developing nematode biocontrol agents (3, 38). This fungus was also the first NTF for which the complete genome was sequenced, rendering it apt to serve as a model organism in studying fungus–nematode interactions (28, 45). So far, proteomics has been employed to investigate the differentially expressed proteins (DEPs) during the production of adhesive mycorrhizal webs (41), and to identify proteins involved in the cell wall during the transition from saprophytic to predatory stages in *A. oligospora* (17). In addition, the expression profile of conidial proteins has also been characterised under ammonia-suppressed conditions (20), and the changes in protein expression in response to benzaldehyde-mitigated inhibitory stress have been explored (21).

Chitin, a crucial component of the nematode body wall and eggshell, provides structural support and protection to the organism (5). It also plays a vital role in maintaining the rigidity of the eggshell and in conferring resistance against external microbial infections (25). Chitinase, an enzyme capable of breaking down chitin and one expressed by NTFs under chitin stimulus, is significant in the process of nematode predation by NTFs, although the regulatory mechanisms of its expression remain unclear (16). Hence, the primary objective of this study was to elucidate the differential protein profile of *A. oligospora* in response to chitin. Accordingly, a proteomic analysis was conducted before and after chitinase induction *via* chitin stimulus, using liquid chromatography with tandem mass spectrometry (LC-MS/MS) to explore the changes in protein expression profiles. Thereby, this research offers fresh insights into the gene expression regulatory mechanisms during nematode infestation in *A. oligospora*.

## Material and Methods

### Strains and culture conditions

*Arthrobotrys oligospora* and the nematode *Caenorhabditis elegans* were maintained in the Key Laboratory of Preventive Veterinary Medicine at Shihezi University. *Arthrobotrys oligospora* was cultured on yeast extract, peptone, sucrose, sodium chloride and asparagine (YPSSA) medium at 28°C and subsequently preserved. *Caenorhabditis elegans* were preserved on nematode growth medium at 20°C. The DH5α strain of *E. coli* and the GS115 strain of *Pichia pastoris*, the yeast selected to express chitinase, were obtained from Beyotime (Haimen, China).

### Cultivation of mycelial cultures of *A. oligospora* induced to produce chitinase by chitin

The mycelium of *A. oligospora* was inoculated onto cornmeal agar medium and cultured in a fungal incubator at 28°C for 10–12 d. Subsequently, the agar block containing the mycelium was sectioned into 0.5 mm × 0.5 mm pieces and transferred into sterilised fresh corn kernel medium for conidial amplification. After 21 d, the conidia were eluted with sterilised distilled water. The conidia were then counted and inoculated into 0.01% soybean peptone liquid medium (final spore concentration of 10^7^/L), and cultured at 28°C for 5–7 d. Upon mycelium formation, prepared colloidal chitin (8) (Solarbio, Beijing, China) was added to achieve a final concentration of 1%, and the culture was incubated at 28°C for 2–3 d. Both pre- and post-induction culture broths were collected and filtered using sterilised filter paper, and the mycelium was washed with sterilised distilled water and stored at 4°C for further use. The induced strain was named AO-e, while the non-induced strain was named AO-c.

### Phenotypic analysis of *A. oligospora* before and after induction of chitinase production by chitin

Briefly, *A. oligospora* was inoculated in YPSSA medium supplemented with colloidal chitin and incubated for 3–5 d to assess mycelial growth rate. Subsequently, spore production and morphology were examined using an inverted microscope. Upon introduction of *C. elegans*, the preyed-upon nematodes were observed and counted every 12 h under an inverted microscope, and the changes in predatory structures and capabilities were further evaluated.

### Extraction and quantification of mycelial protein before and after chitin induction

In brief, samples of 100 mg of *A. oligospora* mycelia taken before chitinase production induction and samples taken after induction were processed by adding 300 μL of Y-PER Yeast Protein Extraction Reagent (Thermo Fisher Scientific, Rockford, IL, USA). The mycelia were then lysed *via* ultrasonication for 60 min at 4°C and by centrifugation at 12,000 × *g* for 10 min. The supernatant was collected for further analysis. Standard solutions were prepared at concentrations of 0, 0.125, 0.25, 0.5, 0.75, 1.0, 1.5, and 2.0 μg/μL. Subsequently, 25 μL of standards and samples were transferred to an enzyme plate. A 200 μL volume of bicinchoninic acid (BCA) working solution (Thermo Fisher Scientific, Rockford, IL, USA) was added to each well, and the well contents were thoroughly mixed and incubated at 37°C for 40 min. The protein concentration of the samples was determined by measuring the optical density at 562 nm and plotting a standard curve based on these measurements.

### Trypsin digestion

In brief, the protein sample (50 μg) and 50 mM NH_4_HCO_3_ (50 μL) were added to a new Eppendorf tube at a 1 : 1 ratio. Subsequently, dithiothreitol was incorporated to achieve a final concentration of 10 mM and incubated for 60 min in a water bath at 56°C. Iodacetamide was added to make a final concentration of 50 mM and the reaction was allowed to proceed for 45 min shielded from light. Next, precooled acetone was introduced and the solution was stored at –20°C overnight. On the following day, a low-temperature centrifugation was performed for 15 min and the supernatant was discarded. Then the precipitate was resolubilised in NH_4_HCO_3_, trypsin was added in a 100 : 1 enzyme-to-substrate ratio and the solution was digested at 37°C overnight. Following digestion, the peptides were desalted using a custom-made desalting column, and the solvent was removed with a vacuum centrifugal concentrator at 45°C. Finally, the solution was vortexed with 0.1% formic acid and centrifuged at a low temperature, and the supernatant was transferred to an autosampler vial for subsequent mass spectrometry analysis.

### Liquid chromatography–tandem mass spectrometry analysis

The proteomic study of predatory nematode fungi was performed as described by Andersson *et al*. (1). Briefly, the samples were separated using a Vanquish liquid chromatography system (Thermo Fisher Scientific, Waltham, MA, USA). Subsequently, they underwent mass spectrometry analysis on an electrospray-coupled ion trap Orbitrap mass spectrometer (Thermo Fisher Scientific, Waltham, MA, USA). The raw mass spectrometry data were processed using MaxQuant software version 1.6.2.10 (34) to match the UniProt *A. oligospora* (species) database for identification (35). The analysis parameters were set as follows: carbamidomethyl as a fixed modification, oxidation and acetyl (protein N-term) as variable modifications, trypsin as the enzyme, a maximum of two missed cleavage sites, mass errors for both primary and secondary mass spectrometry set to 20 ppm, monoisotopic mass for peptide/fragmentation ions and a significance threshold set at P-value = 0.01.

### Identification and bioinformatics analysis of differentially expressed proteins

Fold change (FC) values ≥2 and ≤0.5 were set as the criteria to compare and identify DEPs in *A. oligospora* before and after induction of chitinase production by chitin. The OmicsBean software (Geneforhealth, Shanghai, China) was employed for the Gene Ontology (GO) functional annotation of these proteins, while Kobas 3.0 (4) was used for Kyoto Encyclopedia of Genes and Genomes (KEGG) enrichment analysis.

### Heterologous expression of differentially expressed chitinase

Total RNA was extracted from the mycelium of *A. oligospora* using an E.Z.N.A. Fungal RNA Mini Kit for preparation (Omega Bio-tek, Norcross, GA, USA) and reverse-transcribed into cDNA with a PrimeScript RT Reagent Kit (TaKaRa Bio, Kusatsu, Japan). Specific primers were used to amplify the differentially expressed chitinase genes, and these genes were subcloned into the pPIC9K vector to generate the recombinant expression vector, pPIC9K-AO-chitinase. The vector was then linearised with *Sac* I and transformed into *Pichia pastoris* GS115 competent cells *via* electroporation. Subsequently, the positive clones were identified through PCR and double digestion, with expression induced by methanol at a final concentration of 1.25%. After this, the recombinant proteins were detected and analysed using 10% sodium dodecyl sulphate–polyacrylamide gel electrophoresis (SDS-PAGE) and Western blot. The primary antibody used was mouse anti-*Arthrobotrys oligospora*–positive serum at a dilution of 1 : 1,200, and horseradish peroxidase–labelled sheep anti-mouse IgG was employed as the secondary antibody at a dilution of 1 : 3,000 (Solarbio).

### Determination of the degradation activity of chitinase against nematodes and their eggs

Recombinant chitinase expressed by *Pichia pastoris* was purified *via* nickel column affinity chromatography as described previously (12). A 400 μL aliquot of recombinant enzyme (4.38 mg/mL) was combined with 100 μL of a suspension containing approximately 1,000 *C. elegans* adults, larvae and eggs, the pH was adjusted to 7.0, and the mixture was subsequently incubated at 28°C. The integrity of the nematode body wall and the structure of the egg shell were periodically assessed under an inverted microscope. Additionally, recombinant chitinase was subjected to a 10-min incubation at 100°C to serve as the control group.

### Statistical analysis of data

The experimental data were statistically analysed using GraphPad Prism 9.5.0 software (GraphPad Software, San Diego, CA, USA). Data were plotted into the required graphs, and the significance of differences was analysed using the *t*-test.

## Results

### Phenotypic analyses of *A. oligospora* before and after chitin interaction

**Fig. 1.**
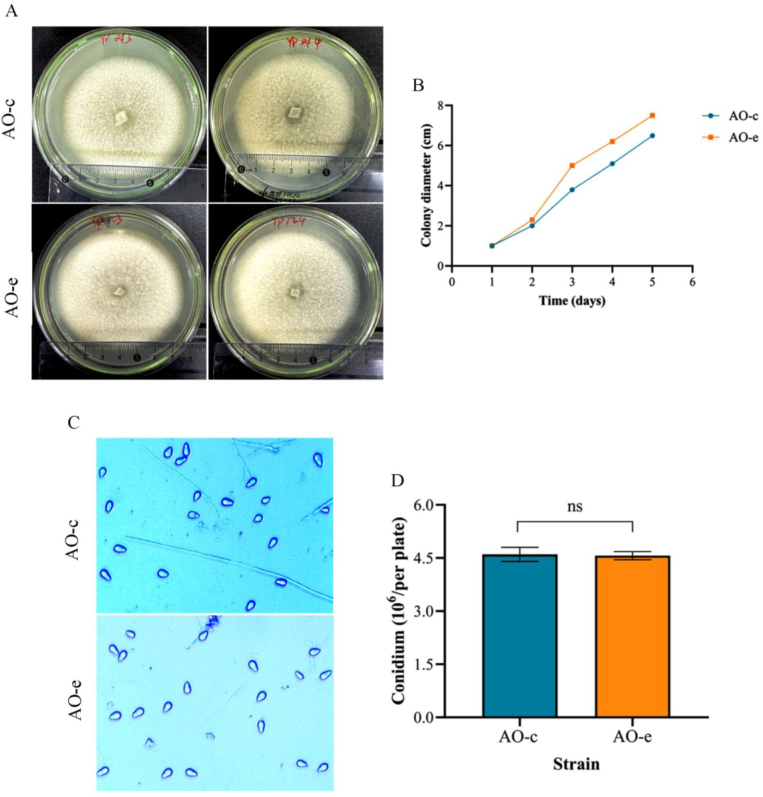
Comparison of the growth rate and sporulation capacity of mycelia in *Arthrobotrys oligospora* before and after chitin interaction. A and B – the colony morphology and its mycelial growth rate; C and D – sporulation ability. AO-c – control group; AO-e – experimental group

**Fig. 2.**
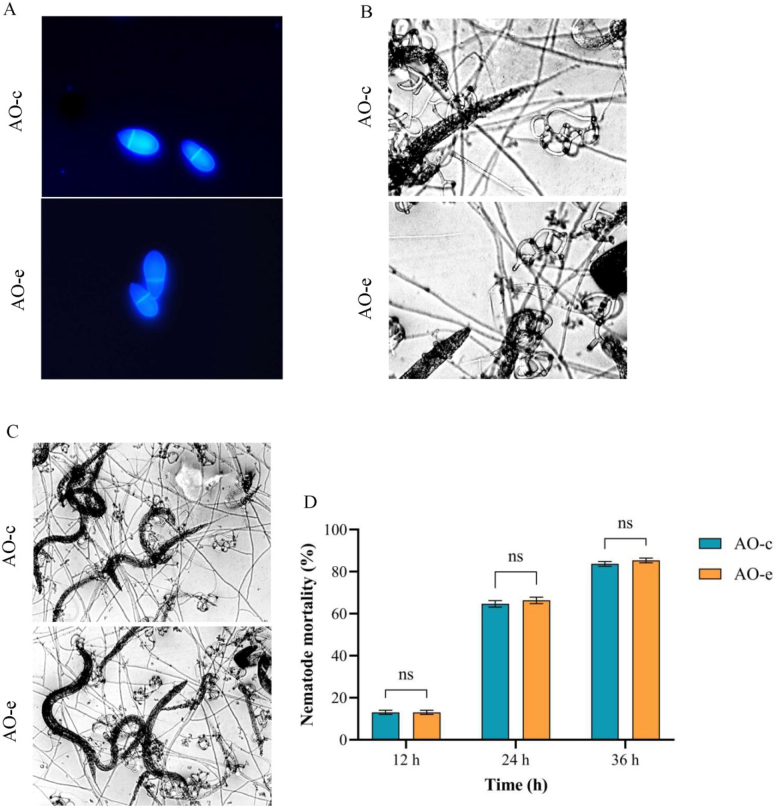
Comparison of morphology, predation structure and predation ability of spores in *Arthrobotrys oligospora* before and after chitin interaction. A – spore morphology; B – predation structure; C and D – predation ability on nematodes. AO-c – control group; AO-e – experimental group

Compared with the control group (AO-c), the growth rate of the group which interacted with chitin (AO-e) was significantly accelerated (Fig. 1 A and 1 B). There were no significant changes in spore production ability (Fig. 1 C and 1 D), spore morphological structure (Fig. 2 A), predatory structure (Fig. 2 B) or nematode predation ability (Fig. 2 C and 2 D).

### Mycelial proteome identification before and after chitin interaction

As shown in Supplementary Fig. 1, the standard curve for the BCA assay was established with the equation y = 869.21x – 78.487, where x is the optical density at 562 nm, and an R^2^ of 0.9917. The protein concentration in group AO-c was determined to be 830.71 μg/mL, while it was 704.67 μg/mL in group AO-e. Based on the LC-MS/MS data, 1,216 proteins were identified from 2,985 peptides in the AO-c group, while 455 proteins were identified from 889 peptides in the AO-e group. A total of 307 proteins overlapped in the protein pools of both the AO-c and AO-e groups.

### Identification and bioinformatics analysis of differentially expressed proteins before and after interaction with chitin

Based on the FC threshold of ≥ 2 and ≤0.5, 1,124 DEPs were identified, comprising 183 upregulated and 941 downregulated ones. In the non-overlapping protein set, 161 proteins were upregulated and 909 were downregulated. The top 10 upregulated DEPs based on protein score, the three most highly expressed of which were encoded by the AOL_s00078g37, AOL_s00109g205 and AOL_s00170g93 genes, are listed in Table 1. The top 10 downregulated DEPs based on protein score, the three most highly expressed of which were encoded by the AOL_s00007g459, AOL_s00173g295 and AOL_s00079g186 genes, are presented in Table 2. Within the intersecting protein group, 54 proteins displayed differential expression, comprising 22 upregulated and 32 downregulated proteins ones. The highest-ranking 10 proteins among the upregulated ones and the equivalent among the downregulated proteins according to FC are documented in Table 3 and Table 4, respectively.

**Table 1.** The top 10 proteins with differentially upregulated expression among non-intersection proteins of *Arthrobotrys oligospora* after chitin interaction

Protein ID	Protein name	Gene name	Score
G1X8M1	Ubiquitin-like domain-containing protein	AOL_s00078g37	323.31
A0A8H8UWC8	V-type proton ATPase subunit	AOL_s00109g205	313.43
A0A7C8N5S6	Superoxide dismutase	AOL_s00170g93	277.7
G1XBG1	Ribosomal protein S15	AOL_s00078g258	266.86
G1XJC0	AA1-like domain-containing protein	AOL_s00097g447	173.69
A0A7C8KEK2	Copper acquisition factor BIM1-like domain-containing protein	EYR41_002241	147.12
A0A8H2E0I4	Neutral ceramidase	AOL_s00076g455	141.71
A0A7C8NSM9	Uncharacterised protein	AOL_s00076g540	109.37
G1XJ38	Ribosomal protein S7 domain-containing protein	AOL_s00097g365	99.319
G1WY88	SAC domain-containing protein	AOL_s00004g248	72.201

1 V-type – vacuolar-type; ATPase – adenosine triphosphatase; AA1 – Alt a 1 protein; BIM-1 – microtubule binding protein 1; SAC – spindle assembly checkpoint

**Table 2.** The top 10 proteins with differentially downregulated expression among non-intersection proteins of *Arthrobotrys oligospora* after chitin interaction

Protein ID	Protein name	Gene name	Score
A0A7C8UKJ8	GH16 domain-containing protein	AOL_s00007g459	323.31
A0A7C8N8K3	Ribonuclease II/R domain-containing protein	AOL_s00173g295	323.31
G1XD83	Sla2 Src-like adaptor 2	AOL_s00079g186	303.54
G1XIU1	Glycosyl transferase family 1 domain-containing protein	AOL_s00097g268	275.82
A0A7C8NE69	Aminotransferase class V domain-containing protein	TWF102_003885	239.24
G1XCR6	Hsp70 protein that interacts with Zuo1p	AOL_s00079g236	231.82
A0A6G1M536	Uncharacterised protein	AOL_s00083g273	226.37
A0A7C8P375	Tubulin alpha chain	AOL_s00078g243	224.51
A0A7C8UJ53	3-hydroxyacyl-CoA dehydrogenase NAD binding domain-containing protein	AOL_s00110g113	219.76
G1WXJ7	Uracil phosphoribosyl transferase	AOL_s00004g7	198.86

1 GH16 – glycoside hydrolase family 16; Sla2 – synthetic lethal acting-binding adaptor protein 2; Src – sarcoma (proto-oncogene tyrosine-protein kinase); Hsp70 – heat-shock protein 70; Zuo1p – zuotin 1 protein; CoA – coenzyme A; NAD – nicotinamide adenine dinucleotide

**Table 3.** The top 10 proteins with differentially upregulated expression among intersection proteins of *Arthrobotrys oligospora* after chitin interaction

Protein ID	Protein name	Gene name	Fold change
A0A7C8NPA7	Chitinase	AOL_s00006g492	25.19742557
G1XLE5	Peptide hydrolase	AOL_s00110g315	13.27323346
A0A7C8UMP1	Beta-hexosaminidase	AOL_s00080g286	11.49963777
A0A7C8PQM7	Aminotransferase class I/class II domain-containing protein	AOL_s00004g264	7.175107854
A0A7C8NRM1	FAD dependent oxidoreductase domain-containing protein	AOL_s00117g57	6.570614843
G1XG12	Aminotransferase class I/class II domain-containing protein	AOL_s00081g305	5.443764901
A0A7C8NZY3	AA1-like domain-containing protein	AOL_s00043g141	4.887246226
A0A7C8JS84	Urease (fragment)	AOL_s00080g26	4.05366717
A0A7C8K8Z4	Glucose-methanol-choline oxidoreductase C-terminal domain-containing protein	AOL_s00006g571	4.050636221
G1X3Q8	Glucan 1,4-alpha-glucosidase	AOL_s00043g332	3.844588344

1 FAD – flavin adenine dinucleotide; AA1 – Alt a 1 protein

**Table 4.** The top 10 proteins with differentially downregulated expression among intersection proteins of *Arthrobotrys oligospora* after chitin interaction

Protein ID	Protein name	Gene name	Fold change
A0A7C8KHY8	Translation elongation factor IF5A C-terminal domain-containing protein	AOL_s00112g56	0.261838199
A0A7C8N2H5	kinase domain-containing protein	AOL_s00079g254	0.246729316
A0A7C8PAR5	Vacuolar protease A	AOL_s00054g478	0.24519951
G1XG20	60S ribosomal protein L3	AOL_s00081g313	0.238139381
A0A7C8V393	60S ribosomal protein L17	AOL_s00097g411	0.226801505
G1XMA1	Uncharacterised protein	AOL_s00140g70	0.210533753
A0A8H2DUD7	Apple domain-containing protein	AOL_s00017g64	0.174564146
G1XEX5	PBP domain-containing protein	AOL_s00080g360	0.157891296
A0A7C8R9J6	Phosphatidylserine decarboxylase	AOL_s00097g76	0.150443331
A0A7C8NXE9	Translation elongation factor 1-alpha (fragment)	AOL_s00169g48	0.135301915

1 IF5A – initiation factor 5A; PBP – penicillin-binding protein

### Gene Ontology analysis

**Fig. 3.**
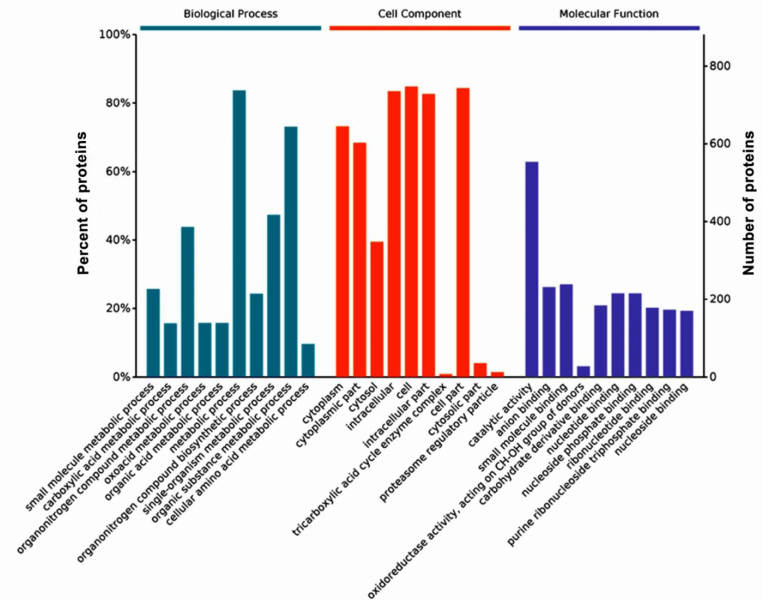
Gene Ontology enrichment analysis of differentially expressed proteins in *Arthrobotrys oligospora* before and after chitin interaction

The GO functional annotation of the DEPs was compared between the AO-c group and the AO-e group (Fig. 3). This study identified 450 biological processes with significant differences. Notably, the 10 most significantly enriched categories revealed that the DEPs were predominantly involved in processes such as carboxylic acid metabolism, keto acid metabolism, organic nitrogen compound biosynthesis, small molecule metabolism containing bases, nucleotide metabolism and small molecule biosynthesis (Supplementary Table 1). There were 114 cellular component categories which were significantly enriched, indicating that the DEPs were primarily localised to cellular structures and organelles (Supplementary Table 2). Additionally, 99 molecular function categories were significantly enriched where the proteins which were DEPs typically exhibited functions related to catalysis and binding, including small molecule binding, oxidoreductase activity, sugar derivative binding, and nucleotide binding (Table 5).

**Table 5.** Gene Ontology molecular function categories that were most significantly enriched in differentially expressed proteins in *Arthrobotrys oligospora* after chitin interaction

Level	Gene Ontology name	Gene Ontology ID	P-value	Count
2	catalytic activity	GO:0003824	5.78E–17	554
4	anion binding	GO:0043168	1.01E–10	232
3	small molecule binding	GO:0036094	4.36E–09	239
4	oxidoreductase activity, acting on CH-OH group of donors	GO:0016614	4.14E–08	28
3	carbohydrate derivative binding	GO:0097367	8.13E–08	184
4	nucleotide binding	GO:0000166	0.000000215	216
4	nucleoside phosphate binding	GO:1901265	0.000000215	216
4	ribonucleotide binding	GO:0032553	0.000000422	178
5	purine ribonucleoside triphosphate binding	GO:0035639	0.00000052	173
4	nucleoside binding	GO:0001882	0.000000604	170

### Kyoto Encyclopedia of Genes and Genomes analysis

**Fig. 4.**
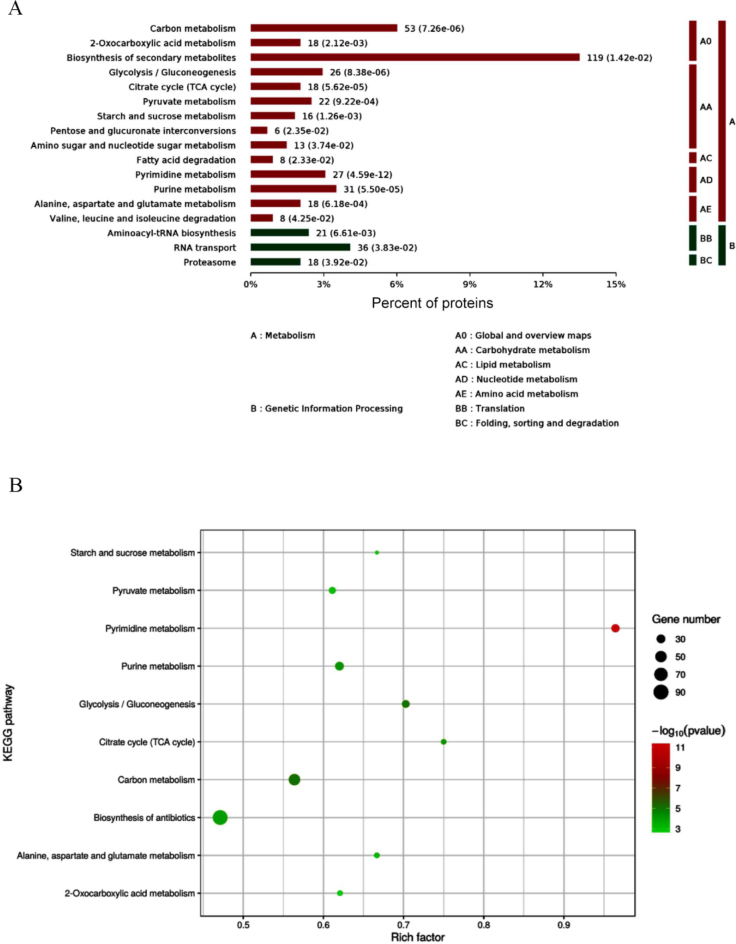
Kyoto Encyclopedia of Genes and Genomes (KEGG) pathway enrichment analysis of differentially expressed proteins (DEPS) in *Arthrobotrys oligospora* before and after chitin interaction. A – KEGG pathway enrichment category of DEPs; B – bubble map of DEPs involved in metabolic pathways in KEGG analysis

**Fig. 5.**
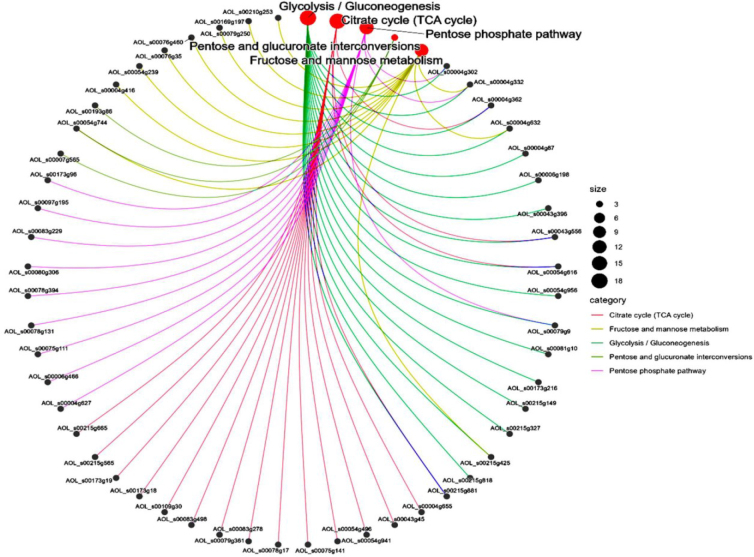
Network diagram analysis of differentially expressed proteins involved in metabolic pathways in *Arthrobotrys oligospora* before and after chitin interaction

A total of 63 significantly different signalling pathways were identified in KEGG pathway analysis (Fig. 4 A). The ten most significantly enriched pathways included pyrimidine metabolism, citric acid cycle, glycolysis, metabolism of alanine, aspartate and glutamate, metabolism of starch and sucrose, 2-oxocarboxylic acid metabolism, pyruvate metabolism, purine metabolism, carbon metabolism and the biosynthesis pathway of antibiotics (Fig. 4 B). Among these, the metabolic pathway was associated with 248 differential proteins, while the biosynthetic pathway of secondary metabolites accounted for 119 differential proteins. The DEPs of *A. oligospora* before and after chitin interaction were predominantly involved in metabolic pathways such as the citric acid cycle, fructose and mannose metabolism, glycolysis, interconversion of pentose and glucuronides and the pentose phosphate pathway (Fig. 5).

### Analysis of differentially expressed chitinases

Screening of known chitinases in *A. oligospora* revealed three significantly differentially expressed proteins. Notably, the expression levels of chitinases AOL_s00004g379 (AO-379) and AOL_s00006g492 (AO-492) were upregulated, while the expression of AOL_s00140g14 (AO-14) was significantly downregulated (Supplementary Table 3). Gene Ontology analysis indicated that these chitinases were involved in chitin catabolism and exhibited hydrolytic enzyme activity, chitinase activity and chitin-binding function. Kyoto Encyclopedia of Genes and Genomes analysis demonstrated that the differentially expressed chitinase participated in carbohydrate metabolism.

### Degradation activity of chitinase AO-379 on nematodes and eggs

The recombinant plasmid pPIC9K-AO-379 was constructed by sequencing and amplifying the gene AO-379 from *A. oligospora* AOL_s00004g379. It was revealed in SDS-PAGE analysis that the molecular weight of the recombinant protein ReAO-379 was approximately 44 kDa, which was consistent with the expected bands (Supplementary Fig. 4 A). Western blot results demonstrated that the protein specifically bound to mouse anti-*Arthrobotrys oligospora* serum (Supplementary Fig. 4 B), confirming the successful expression of the ReAO-379.

**Fig. 6.**
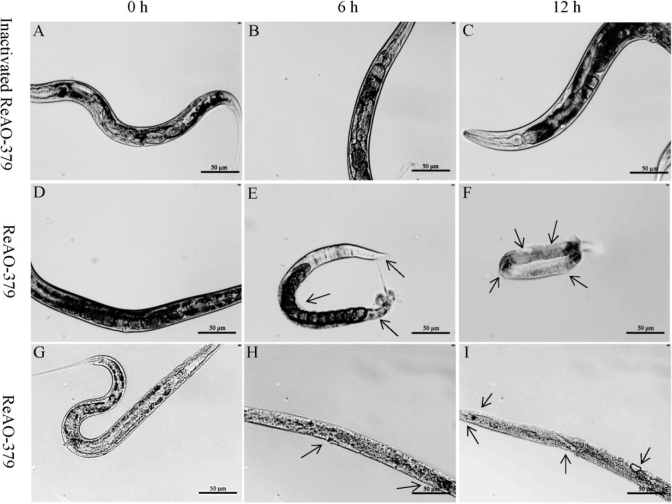
Determination of the degradation activity of differentially expressed chitinase AO-379 in *Caenorhabditis elegans*. A–C – *C. elegans* treated with inactivated ReAO-379 for 0, 6 and 12 h, respectively; D–F – adult *C. elegans* treated with ReAO-379 for 0, 6 and 12 h, respectively; G–I – stage-IV larvae of *C. elegans* treated with ReAO-379 for 0, 6 and 12 h, respectively. Arrow – degraded part of the nematode body wall; scale bar – 50 μm

The body wall of the control group of adult *C. elegans* preserved its smooth and intact appearance throughout the 12-h period, with no morphological alterations observed (Fig. 6 A–C). At 6 h post ReAO-379 treatment, the body wall of adult *C. elegans* remained relatively intact, with notable degradation beginning primarily in the abdominal body wall. However, by 12 h, the body wall exhibited significant crumpling and degradation, and the unhatched eggs were also destroyed and degraded (Fig. 6 D–F). Stage IV *C. elegans* larvae body walls had become blurred and showed slight degradation by the 6^th^ h, and had become pronouncedly crumpled, ruptured and degraded by the 12^th^ h (Fig. 6 G–I).

**Fig. 7.**
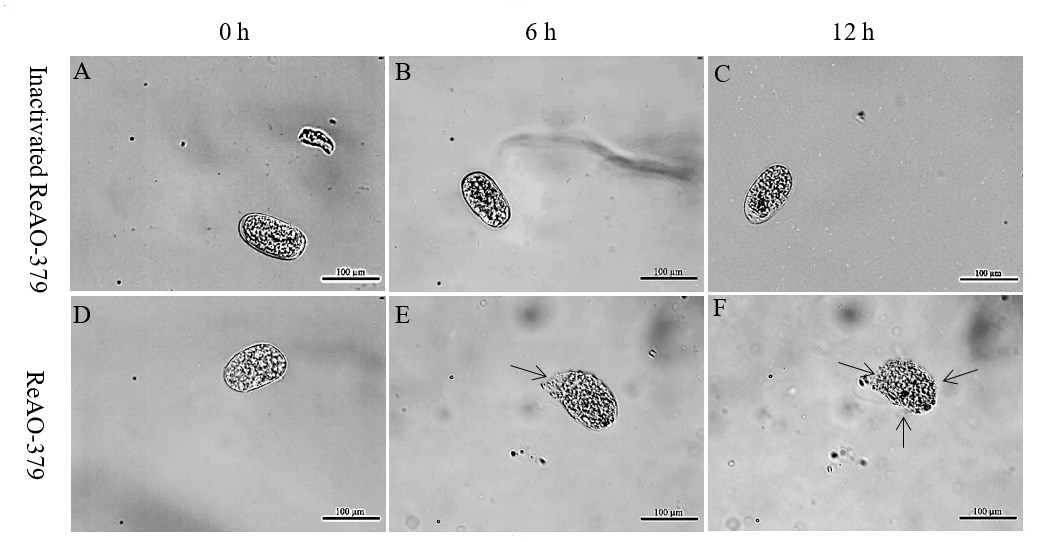
Analysis of the degradation activity of differentially expressed chitinase AO-379 in *Caenorhabditis elegans* eggs. A–C – *C. elegans* eggs treated with inactivated recombinant ReAO-379 for 0, 6 and 12 h, respectively; D–F – *C. elegans* eggs treated with ReAO-379 for 0, 6 and 12 h, respectively. Arrow – degraded part of egg shell; scale bar – 100 μm

The eggshells exposed only to inactivated recombinant chitinase (the control group eggshells) maintained a clear and intact structure with no morphological changes observed (Fig. 7 A–C). When the eggs of *C. elegans* were exposed to ReAO-379 for 6 h, their shells exhibited partial degradation, with some eggs showing noticeable structural distortions and alterations. After 12 h, the surface structure of the eggshell had completely vanished, revealing the contents of the eggs (Fig. 7 D–F).

## Discussion

Chitin, an N-acetyl-D-glucosamine polymer, stands out as one of the most abundant polysaccharides in nature, predominantly located in the exoskeletons of arthropods and the cell walls of fungi (6, 24, 30, 32). Research indicates that chitin constitutes a critical component of the nematode body wall and eggshells, significantly contributing to the integrity and protection of nematodes and their eggs (2, 26, 39). In the present study, we observed a notable increase in the mycelial growth rate following the introduction of colloidal chitin into the medium in *A. oligospora*. This finding suggests that chitin may serve as a polysaccharide resource in fungal cultures, providing an essential source of energy and carbon for mycelial development.

Additionally, proteomic analysis was conducted to identify the protein profiles in *A. oligospora* before and after colloidal chitin interaction. The DEPs as a result of chitin stimulus of the fungus were identified and analysed. Gene Ontology analysis revealed that these proteins predominantly functioned in cellular metabolism. Kyoto Encyclopedia of Genes and Genomes enrichment analysis indicated that the DEPs were primarily involved in carbon metabolism pathways, including the citric acid cycle, glycolysis, and the metabolism of starch, sucrose, amino sugars, ribose, purines and pyrimidines. Notably, KEGG enrichment found that there were also some DEPs involved in the mitogen-activated protein kinase–signalling pathway. These findings suggest that *A. oligospora* responds to an external chitin stimulus by activating signal transduction pathways, which in turn initiate a series of downstream reactions. This leads to rapid adjustments in gene expression, thereby altering metabolic and energy processes, and ultimately supports the organism’s growth and development post-stimulus.

In *A. oligospora*, Yang *et al*. (41, 42) identified 16 chitinase genes through whole genome sequence analysis. These enzymes can be categorised under the glycoside hydrolase 18 family, which are enzymes equipped with conserved substrate binding and catalytic domains, enabling them to catalyse the degradation of chitin’s β-1,4 glycosidic bonds. It has been observed that environmental variations significantly influence the expression levels of chitinase in *A. oligospora* (42). Notably, the absence of chitin significantly alters the expression of various chitinases in NTF (33), indicating that chitinase expression is regulated by environmental chitin concentrations. This study reveals that the differential expression of chitinase AO-379 and AO-492 is upregulated, implying their crucial roles in the infestation of nematodes by *A. oligospora*. Furthermore, the recombinant chitinase AO-379 expressed in *Pichia* yeast demonstrated substantial degradation activity against *C. elegans* and its eggs, affirming its potent effect on nematode body walls and eggshells. The results of this study also provide a new experimental basis for *A. oligospora* biomaterial in the further development of efficient and environmentally friendly control agents for gastrointestinal nematodiasis in livestock. We may be able to express a large amount of chitin-induced upregulated chitinase and prepare a biological agent, namely a chitinase preparation, for the prevention and treatment of gastrointestinal nematodes in livestock.

Related studies have indicated that β-hexosaminidase is extensively distributed across fungi and is co-expressed with chitinase, and plays a crucial role in cell wall chitin metabolism and the utilisation of chitin-containing substrates (22, 27). In this study, β-hexosaminidase expression was significantly up-regulated in *A. oligospora* following chitin induction. This upregulation presumably aids chitinase in further hydrolysing the oligosaccharides decomposed by chitinase to produce N-acetylglucosamine, thereby serving as a carbon source to enhance mycelial growth and development. Consequently, this enzyme likely plays a vital auxiliary role in the penetration of the nematode body wall cuticle by *A. oligospora*. Perhaps the enzyme can also be investigated as an auxiliary direction for the development of chitinase biological agents. Additionally, proteomic analysis of proteins differentially expressed upon chitin stimulus revealed that the upregulated proteins were predominantly hydrolytic enzymes and catalase. This suggests that *A. oligospora* can swiftly regulate the expression of metabolism-related genes in response to external chitin stimulus. Nonetheless, the key molecules and signalling pathways activated in response to external chitin warrant further investigation.

## Conclusion

This study for the first time unveiled the expression profiles of chitin-responsive proteins in *A. oligospora*. It demonstrated that *A. oligospora* could regulate its metabolic reactions and physiological activities through the modulation of the gene expression of metabolic enzymes related to glucose metabolism, hydrolases and catalases, which provides new insights into the molecular mechanism of nematode predation in *A. oligospora*.

## Supplementary Material

Supplementary Material Details

Supplementary Material Details
