# Supplementary material for: Proteomic insights into nematode-trapping fungi Arthrobotrys oligospora after their response to chitin
Source: J Vet Res. 2025 Feb 25;69(1):71–82. doi: 10.2478/jvetres-2025-0005 (PMC11936082; doi:10.2478/jvetres-2025-0005)
Supplement: Supplementary file 1 — Supplementary Material Details [file jvetres-2025-0005_sm1.pdf]

Supplementary Table 1. The 10 most significantly entries in the Biological Process classification of differentially expressed proteins in *Arthrobotrys oligospora* in Gene Ontology (GO) enrichment analysis

Levels	GO Name	GO ID	P-value	Count
4	small molecule metabolic process	GO:0044281	7.76E−24	227
6	carboxylic acid metabolic process	GO:0019752	2.26E−18	138
4	organonitrogen compound metabolic process	GO:1901564	3.43E−18	386
5	oxoacid metabolic process	GO:0043436	1.49E−17	139
4	organic acid metabolic process	GO:0006082	2.06E−17	139
2	metabolic process	GO:0008152	1.38E−13	738
5	organonitrogen compound biosynthetic process	GO:1901566	5.04E−12	215
3	single-organism metabolic process	GO:0044710	1.37E−11	417
3	organic substance metabolic process	GO:0071704	6.33E−11	644
4	cellular amino acid metabolic process	GO:0006520	6.52E−11	85

Supplementary Table 2. The 10 most significantly items in the Cellular Component classification of differentially expressed proteins in *Arthrobotrys oligospora* in Gene Ontology (GO) enrichment analysis

Levels	GO Name	GO ID	P-value	Count
4	cytoplasm	GO:0005737	1.8E-14	645
4,v	cytoplasmic part	GO:0044444	3.68E-13	603
5,6	cytosol	GO:0005829	2.23E-11	348
3	intracellular	GO:0005622	0.00000141	736
2	cell	GO:0005623	0.00000158	748
3	intracellular part	GO:0044424	0.00000209	729
4	tricarboxylic acid cycle enzyme complex	GO:0045239	0.0000039	8
2	cell part	GO:0044464	0.00000506	744
5,6,7	cytosolic part	GO:0044445	0.0000171	36
4	proteasome regulatory particle	GO:0005838	0.0000271	13

Supplementary Table 3. Differentially expressed chitinase in *Arthrobotrys oligospora* after chitin interaction

Protein IDs	Protein names	Gene names	Score	LFQ intensity
A0A7C8P479	GH18 domain-containing protein; Chitinase	AOL_s00004g379	7.3446	43287000
A0A7C8NPA7	Chitinase	AOL_s00006g492	33.726	1276300000
G1XM45	Chitinase	AOL_s00140g14	19.798	0
