# Supplementary material for: Proteomic insights into nematode-trapping fungi Arthrobotrys oligospora after their response to chitin
Source: J Vet Res. 2025 Feb 25;69(1):71–82. doi: 10.2478/jvetres-2025-0005 (PMC11936082; doi:10.2478/jvetres-2025-0005)
Supplement: Supplementary file 2 — Supplementary Material Details [file jvetres-2025-0005_sm2.pdf]

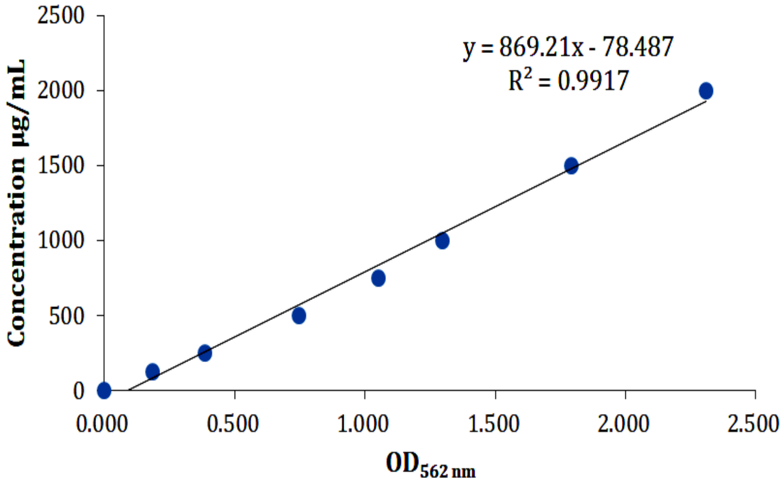

Supplementary Fig. 1. The established standard curve of the bicinchoninic acid protein assay used to quantify protein concentrations in the nematode-trapping fungus *Arthrobotrys oligospora* mycelia before and after chitin interaction

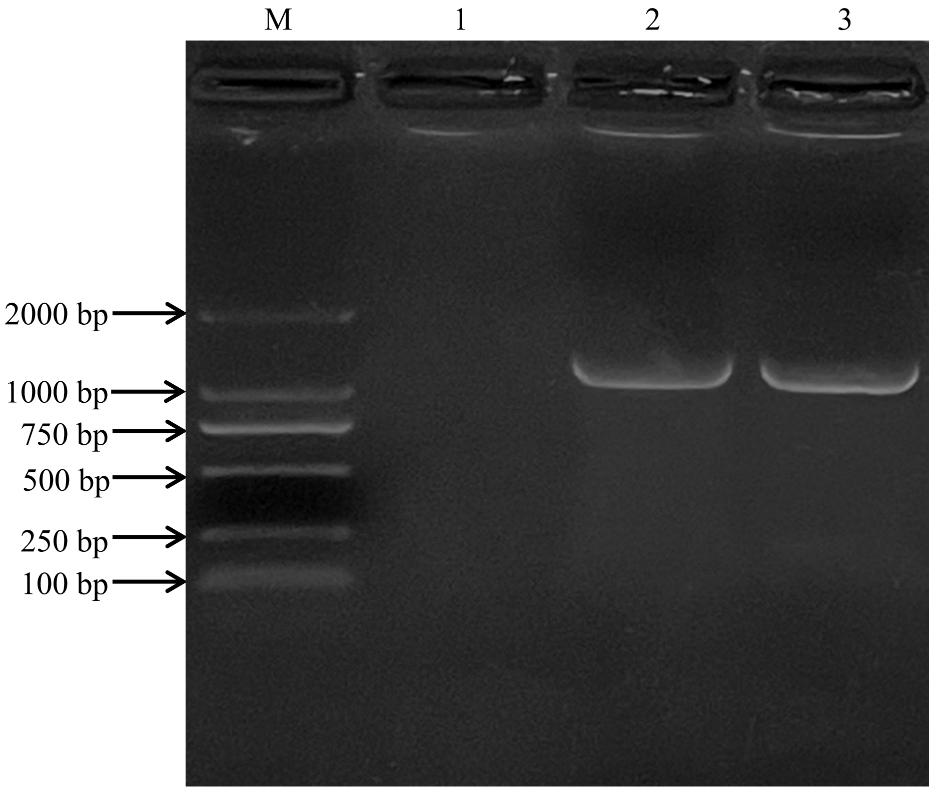

Supplementary Fig. 2. PCR amplification of differentially expressed chitinase AO-379 after chitin interaction M – DL-2000 DNA marker; 1 – negative control; 2–3 – amplified fragment of the AO-379 gene

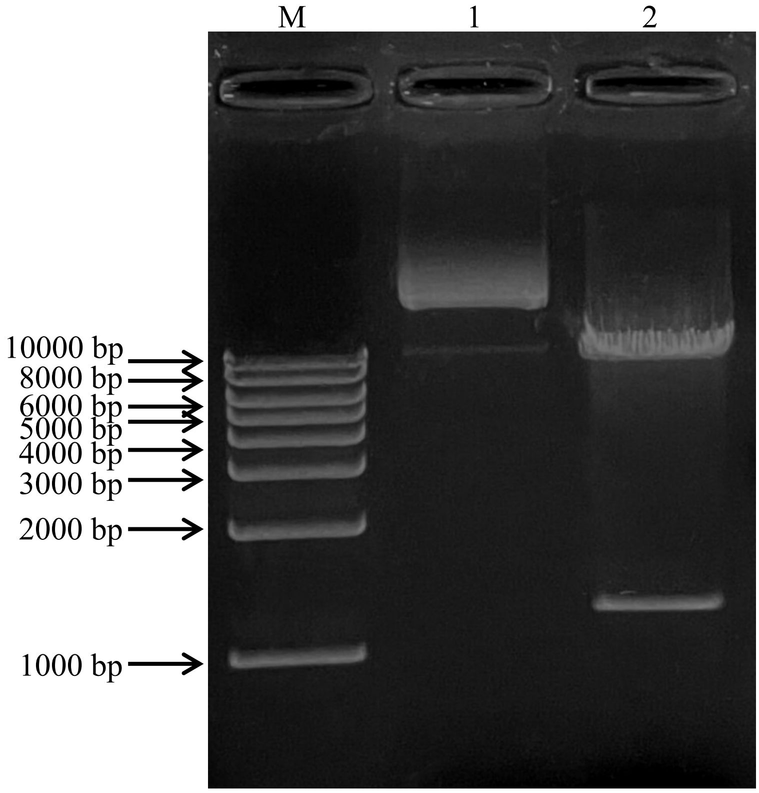

Supplementary Fig. 3. Identification of recombinant plasmid pPIC9K-*Arthrobotrys oligospora* (AO)-379 by double-enzyme digestion

M – DL-10000 DNA Marker; 1 – plasmid pPIC9K-AO-379; 2 – plasmid pPIC9K-AO-379 after undergoing double-enzyme digestion

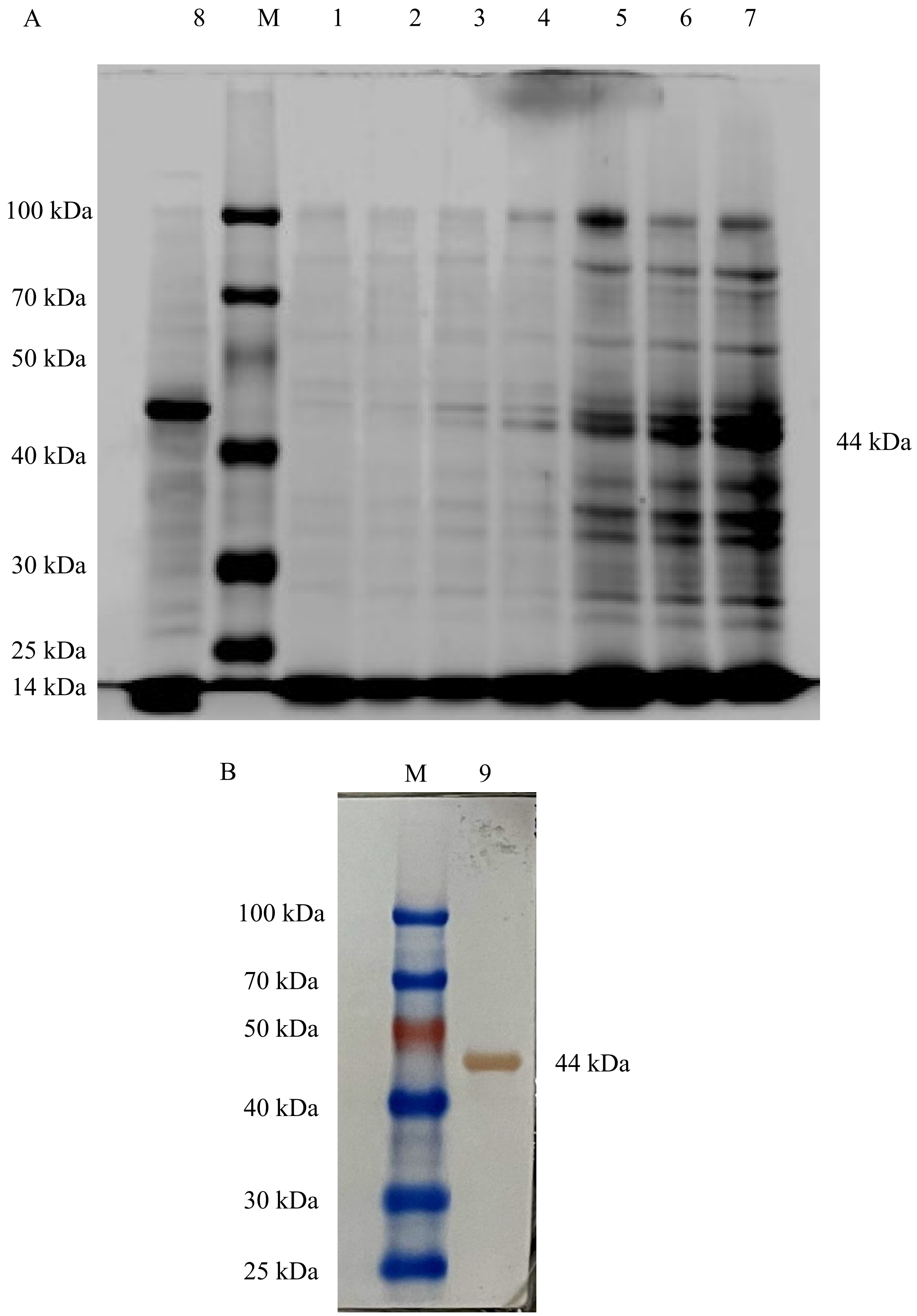

Supplementary Fig. 4. Heterologous expression of differentially expressed chitinase *Arthrobotrys oligospora* (AO)-379. A – Analysis of expressed recombinant protein ReAO-379 by sodium dodecyl sulphate–polyacrylamide gel electrophoresis. M – standard protein molecular; 1 – pPIC9K vector; 2–7 – culture supernatants induced by methanol for 0, 24, 48, 72, 96 and 120 h, respectively; 8 – Purified ReAO-379 protein. B – Identification of ReAO-379 by Western blot
